# Suppressed paraoxonase-1 activity associates with elevated oxylipins and the presence of small airways disease in patients with rheumatoid arthritis

**DOI:** 10.1007/s10067-022-06375-w

**Published:** 2022-09-22

**Authors:** Amir A. Razmjou, Jennifer M. Wang, Ani Shahbazian, Srinivasa Reddy, Christina Charles-Schoeman

**Affiliations:** David Geffen School of Medicine, University of California, Los Angeles, 1000 Veteran Ave, Room 31-79, Los Angeles, CA 90095-1670 USA

**Keywords:** Paraoxonase-1, Rheumatoid arthritis, Small airways disease

## Abstract

**Background:**

Rheumatoid arthritis (RA)-associated lung disease (LD) associates with significantly increased morbidity and mortality. Although oxidative stress plays an important role in the inflammatory responses in other forms of lung disease, minimal work has evaluated its role in RA-LD. The current work examines the relationship between the anti-oxidant HDL-associated enzyme paraoxonase-1 (PON1), the PON1 Q192R polymorphism, and a targeted oxylipin panel with RA-LD.

**Methods:**

This study was conducted as a retrospective chart review of a longitudinal single-center cohort of 250 RA patients. CT scans of the chest were reviewed by the interpreting radiologist and classified as small airways disease (SAD), interstitial lung disease (ILD), and bronchiectasis. PON1 activity was measured by its lactonase, arylesterase, and paraoxonase functions. The PON1 Q192R polymorphism and a targeted lipidomics panel were performed as previously reported.

**Results:**

43.2% of the 250 RA patient cohort (*n* = 108) had available CT scans, including 48 patients (44.4%) with SAD, 27 patients (25.0%) with bronchiectasis, and 16 patients (14.8%) with ILD. Patients with SAD had significantly lower baseline PON1 activity by its arylesterase, and lactonase functions, as well as higher 15-HETE, LTB4, and PGE2 levels compared to those without SAD. These predictors of SAD remained significant after multivariate analysis including known risk factors for RA-LD. Suppressed PON1 activity also correlated with higher levels of 15-HETE and 12-HETE.

**Conclusion:**

In a single-center RA cohort, suppressed baseline PON1 activity and elevation in the oxylipins 15-HETE, LTB4, and PGE2 predicted the presence of RA-SAD in longitudinal follow-up.

**Table:** 

**Key Points** *• Small airways disease (SAD) was present in 44.4% of this rheumatoid arthritis (RA) cohort.* *• Patients with SAD had significantly lower baseline PON1 activity, as well as higher levels of the oxylipins 15-HETE, LTB4, and PGE2 levels compared to those without SAD.* *• Further work is warranted to confirm these findings and further define the role of PON1 and lipid oxidation in RA lung disease.*

**Supplementary Information:**

The online version contains supplementary material available at 10.1007/s10067-022-06375-w.

## Introduction

Rheumatoid arthritis (RA) is the most common autoimmune arthropathy, with an estimated prevalence of 0.5–1%, and with significant burden on health-related quality of life and disability [1]. While RA is characterized predominantly by an erosive inflammatory arthritis, it is a systemic disease, which may include extra-articular manifestations such as RA-associated lung disease (RA-LD). RA-LD can affect nearly any compartment of the respiratory tract [2], and includes interstitial lung disease (ILD) with usual interstitial pneumonia (UIP) or non-specific interstitial pneumonia (NSIP), small airways disease (SAD), bronchiectasis, pleural disease, and pulmonary vascular disease [2].

RA-LD, particularly RA-ILD, associates with high morbidity and mortality, and risk factors for RA-ILD include male gender, age, disease duration, seropositivity, obesity, and glucocorticoid use [3]. Several of these risk factors also associate with cardiovascular (CV) risk in RA patients, suggesting possible shared pathogenic mechanisms [4].

Paraoxonase-1 (PON1) is an HDL-associated enzyme which mediates much of high-density lipoprotein’s (HDL) anti-oxidant, anti-inflammatory functions via its paraoxonase, arylesterase, and lactonase activities [5]. PON1 metabolizes pro-inflammatory bioactive lipid mediators (BLM), reducing oxidized low-density lipoprotein (oxLDL) levels and decreasing endothelial cell activation and atherogenesis [6]. In patients with RA, suppressed PON1 activity and the Q192R polymorphism, which affects enzyme activity, have been associated with increased atherosclerotic risk despite “normal” traditional cholesterol levels [7].

In the current work, we examine the relationship between PON1 activity, the PON1 Q192R polymorphism, and a targeted panel of BLM with the development of RA-LD in a large longitudinal RA cohort.

Cardiovascular disease (CVD) is a leading killer of patients with rheumatoid arthritis (RA) who have a marked increase in CV morbidity and mortality compared to the general population [8–10]. Systemic inflammation from active RA strongly associates with CV risk in RA patients [11–13], but mechanisms by which inflammation increases CV morbidity and mortality remain poorly understood. Traditional cholesterol levels may be suppressed in the setting of active RA, further complicating the clinical CV risk assessment.

High-density lipoprotein (HDL) is normally an anti-atherogenic molecule that regulates systemic inflammation by promoting cholesterol efflux and preventing oxidation of low-density lipoproteins (LDL) [14–17]. However, in the setting of acute or chronic inflammation, the HDL particle may be converted to a dysfunctional, even pro-inflammatory particle by alteration in the level and function of several HDL-associated proteins and lipids [18–23]. Our previous work has suggested that abnormal anti-oxidant function of HDL and its associated protein, paraoxonase-1 (PON1), may be a mechanism through which RA-associated inflammation drives CV risk [7, 19, 24].

We recently reported that HDL is increased in synovial fluid of RA patients, which contains elevated levels of pro-inflammatory oxlipins, that associate with impaired anti-oxidant function of the particle [18]. We have also shown an accumulation of pro-inflammatory lipids in HDL particles in circulation, which associate with impaired HDL function [18]. We hypothesize that HDL may become oxidatively modified and dysfunctional in the inflammatory milieu of the RA joint, similar to what has been reported in the vessel wall of patients with coronary artery disease [25].

## Materials and methods

### Study design

A retrospective chart review was performed of a longitudinal single-center cohort of RA patients at the University of California, Los Angeles (UCLA) medical center to identify patients who had available computed tomography (CT) scans of the chest in the electronic medical record at time of review in May 2021. All patients met the American College of Rheumatology 1987 RA classification criteria. Patients provided written informed consent, and the study was approved by the Human Research Protection Program at UCLA. Data on CVD risk and RA disease characteristics were obtained by previously administered questionnaires and chart review. Baseline disease activity was assessed by the disease activity score with 28 joint count (DAS28) using erythrocyte sedimentation rate (ESR).

Baseline specimens of all patients entering the longitudinal cohort were used for laboratory analyses as described below. Traditional lipid profiles and inflammatory markers (high-sensitivity C-reactive protein (hsCRP), and ESR) were measured at the UCLA Clinical Laboratory at the time of the biospecimen blood draw.

### High-resolution computed tomography of the chest

High-resolution CT (HRCT) scans of the chest were interpreted by a thoracic radiologist and categorized with the following features: ILD (UIP, NSIP, other ILD), SAD (peribronchial thickening, air trapping, centrilobular nodules), and bronchiectasis. Pulmonary function tests as performed for usual clinical care were also evaluated and assessed for the following: forced expiratory volume over 1 s percent predicted (FEV1%), forced vital capacity percent predicted (FVC%), FEV1/FVC, diffusing capacity for carbon monoxide percent predicted and corrected for hemoglobin (DLCO%), and forced expiratory flow at 25–75% of FVC percent predicted (FEF25–75%).

### PON1 activity, the PON1 Q192R polymorphism, and lipidomics analyses

Paraoxonase, lactonase, and arylesterase activities of PON1 were measured in patient plasma samples as described previously [26]. The PON1 Q192R genotype (QQ, QR, or RR) was determined using DNA extracted from peripheral blood samples as described previously [7]. Mass spectrographic analysis was performed on a SCIEX 5500 QTrap run in negative ion mode as described previously and controlled by Analyst 1.6.2 software, for a BLM panel including: 20-hydroxy leukotriene B4 (20-OH LTB4), thromboxane B2 (TBX2), prostaglandin E2 (PGE2), 6-trans 12-epi leukotriene B4 (6t12eLTB4), leukotriene B4 (LTB4), 13-hydroxyoctadecadienoic acid (13-HODE), 12-HODE, 9-HODE, 15-hydroxyeicosatetraenoic acid (15-HETE), 12-HETE, 11-HETE, 5-HETE, 14-s hydroxydocosahexaenoic acid (14sHDHA), and 5-oxo-HETE [26].

### Statistical analysis

Data were analyzed using JMP Pro 14.0 (SAS Institute Inc., Cary, NC, USA). Groups were compared using Student’s *t*-test for continuous variables and the chi-square test of association for categorical variables, along with Fisher’s exact test for small sample sizes. When needed, nonparametric Wilcoxon rank-sum tests were used to analyze continuous variables. Correlations between variables were evaluated using the Pearson’s correlation coefficient for normally distributed data, and Spearman’s correlation coefficient for nonparametric data. The significance level was pre-specified at *p* < 0.05.

Initial analyses compared baseline PON1 activities and BLM in univariate analyses between patients with and without major types of lung disease (ILD, SAD, bronchiectasis). Forward multivariate stepwise logistic regression analyses were next performed to evaluate PON1 and BLM as predictors of RA-LD after controlling for known risk factors for lung disease in RA, including RA disease duration, age, sex, smoking, and obesity (body mass index (BMI)). Because of significant correlations between PON1 activities and BLM, individual MV models were used to test the associations of PON1 activities and BLM with lung disease outcomes.

## Results

### Population demographics, clinical characteristics, and CT findings

43.2% of the 250 RA patient cohort (*n* = 108) had available CT scans of the chest for review and were included in the study. These patients were predominantly female (85%), Caucasian (77%), and had chronic RA with a disease duration of 19.7 ± 15.0 years at the time of CT scan (mean ± standard deviation (SD)) (Supplemental Table 1). Small airways disease was present in 48 (44.4%) of patients (Supplemental Fig. 1), which included peribronchial thickening in 43 (39.8%), air trapping in 24 (22.2%), and centrilobular nodules in 10 (9.3%). Patients with SAD had a significantly reduced FEV1/FVC of 71.4 ± 12.4 compared to patients without SAD (*p* = 0.01) (Supplemental Table 2). ILD was present radiographically in 16 patients (14.8%) and bronchiectasis was present in 27 patients (25.0%) (Supplemental Fig. 1). Patients with ILD had a significantly reduced DLCO% of 63.5 ± 27.9 when compared to patients without ILD (*p* = 0.04).

Baseline patient clinical characteristics were similar between patients with and without SAD, ILD, and bronchiectasis (Table 1). Patients had active RA on entry into the cohort with high DAS28 scores and elevation in inflammatory markers (Table 1). Few patients in the cohort had a history of cardiovascular disease at baseline and low numbers of patients were receiving statins, including no patients using statins in the ILD subgroup. Baseline medication use was similar across groups with the exception of TNF inhibitor use which was higher in patients with ILD on longitudinal CT scan compared to patients without ILD (*p* = 0.03). Overall, there were no significant differences in patient characteristics between those with and without SAD, ILD, or bronchiectasis, other than significantly higher TNF inhibitor use in those with ILD. The mean time from the baseline specimen collection to CT scan assessments was 5–6 years across all groups, and was lowest in the bronchiectasis group (Table 1).

**Table 1:** Clinical Characteristics of RA patients stratified by lung disease

	SAD present (*N* = 48)	SAD absent (*N* = 60)	ILD present (*N* = 16)	ILD absent (*N* = 92)	Bronchiectasis present (*N* = 27)	Bronchiectasis absent (*N* = 81)
Age (years)	63 ± 11.0	62 ± 13.4	64 ± 8.4	63 ± 12.9	66 ± 10.6	62 ± 12.7
Female	39(81.3)	53(88.3)	12(75.0)	81 (87.1)	22(81.5)	71(86.6)
Race-Caucasian	38(79.2)	45(75.0)	11(68.8)	72 (77.4)	17(63.0)	66(80.5)
Ethnicity-Hispanic	10(20.8)	14(23.3)	6(37.5)	19(20.4)	7(25.9)	18(22.0)
BMI (kg/m^2^)	29.6 ± 8.3	28.6 ± 6.5	27.5 ± 4.4	29.3 ± 7.7	28.6 ± 6.9	29.2 ± 7.6
RA disease duration prior to CT (years)	23.0 ± 18.0	16.9 ± 11.1	16.0 ± 7.1	20.4 ± 15.9	21.6 ± 21.6	19.1 ± 12.0
Time from specimen collection to CT (years)	6.4 ± 3.7	5.6 ± 3.4	5.2 ± 3.1	6.1 ± 3.6	4.7 ± 3.5**	6.4 ± 3.5
hsCRP (mg/L)	11.0 ± 17.6	6.4 ± 16.2	12.3 ± 25.7	7.8 ± 15.1	9.8 ± 17.7	8.0 ± 16.7
ESR (mm/hour)	29.3 ± 22.1	27.0 ± 20.7	32.7 ± 23.6	27.4 ± 20.8	31.9 ± 19.4	26.9 ± 21.8
DAS28	4.9 ± 1.7	4.5 ± 1.8	4.4 ± 2.0	4.7 ± 1.7	4.4 ± 1.5	4.7 ± 1.8
HAQ-DI	1.0 ± 0.8	1.1 ± 0.8	1.1 ± 1.0	1.1 ± 0.8	1.2 ± 0.9	1.1 ± 0.8
Medications						
Methotrexate	22(47.8)	28(48.3)	4 (26.7)	46(51.1)	10(40.0)	40(50.0)
TNF inhibitor	18(39.1)	25(43.1)	10 (66.7)*	33(36.7)	12(48.0)	31(38.8))
Other biologic	5(11.1)	6(10.9)	1 (6.7)	10(11.6)	3(12.0)	8(10.5)
Prednisone	12(26.1)	22(37.9)	8 (53.3)	26(28.9)	8(32.0)	26(32.5)
Statins	4(8.7)	10(17.2)	0 (0.0)	14(15.6)	3(12.0)	11(13.8)
Cardiovascular risk factors						
H/o myocardial infarction	2(5.1)	2(3.5)	0(0.0)	5(6.0)	3(12.5)	2(2.7)
H/o cerebrovascular accident	2(5.3)	2(3.7)	0(0.0)	5(6.0)	2(8.3)	3(4.1)
Hypertension	23(47.9)	29(48.3)	3(23.1)	40(47.6)	11(45.8)	32(43.8)
Diabetes	4(8.3)	9(15.0)	1(7.7)	10(11.9)	3(12.5)	8(11.0)
Current tobacco use	3(8.1)	2(3.9)	1(7.7)	2(2.4)	1 (4.2)	2(2.7)
Past tobacco use	9(27.3)	13(26.5)	6(46.2)	21(25.0)	9 (37.5)	18(24.7)
Total cholesterol (mg/dL)	187 ± 41.5	200 ± 48.4	210 ± 55.2	191 ± 43.6	194 ± 52.9	193 ± 43.2
LDL cholesterol (mg/dL)	108 ± 31.9	109 ± 43.4	115 ± 54.0	107 ± 35.7	105 ± 40.6	109 ± 37.9
HDL cholesterol (mg/dL)	61 ± 20.4	66 ± 22.1	68 ± 27.0	62 ± 20.5	68 ± 23.3	52 ± 20.7
Triglycerides (mg/dL)	113 ± 70.6	136 ± 100.6	158 ± 152.6	120 ± 73.0	108 ± 47.4	131 ± 98.6

Values are *n*(%) or mean ± SD.*p = 0.03 (TFNi more in ILD); **p = 0.04 (longer from specimen to CT in no bronchiectasis). *SAD* small airways disease, *ILD* interstitial lung disease

### Suppressed baseline PON1 activity associates with presence of small airways disease on longitudinal follow-up

Patients with SAD on CT scan had significantly lower baseline PON1 activity as measured by both arylesterase and lactonase activities of the enzyme compared to patients without SAD on CT scan (Table 2). No differences in traditional lipid measures including high-density lipoprotein cholesterol (HDL-C), low-density lipoprotein cholesterol (LDL-C), total cholesterol and triglyceride levels, the PON1 Q192R polymorphism, or RA disease activity measures at baseline including DAS28, ESR, or hsCRP were noted between patients with and without SAD (Table 1).

**Table 2:** PON1 activity and Q192 polymorphisms by presence of RA lung disease

	SAD present	SAD absent	ILD present	ILD absent	Bronchiectasis present	Bronchiectasis absent
PON1 Activity (U/ml)						
Arylesterase activity	175.8 ± 63.8*	213.4 ± 78.5	198.2 ± 82.0	196.9 ± 73.3	182.6 ± 70.4	201.9 ± 75.2
Lactonase activity	18.9 ± 8.5*	23.7 ± 12.4	22.4 ± 11.5	21.6 ± 11.1	19.7 ± 10.2	22.4 ± 11.3
Paraoxonase activity	470.4 ± 461.5	443.2 ± 303.0	493.1 ± 287.7	451.7 ± 393.6	394.5 ± 312.5	478.6 ± 398.1
PON-1 Q192R polymorphism^#^						
QQ genotype	16(34.8)	21(35.0)	4(25.0)	33(36.3)	7(26.9)	30(37.0)
QR genotype	19(41.3)	30(50.0)	9(56.3)	40(44.0)	13(50.0)	36(44.4)
RR genotype	11(23.9)	9(15.0)	3(18.8)	18(19.8)	6(23.1)	15(18.5)
Oxylipins (ng/ml)						
20-OH LTB4	0.3 ± 0.2	0.2 ± 0.2	0.3 ± 0.2	0.2 ± 0.2	0.3 ± 0.2	0.2 ± 0.2
TXB2	21.4 ± 26.7	17.1 ± 20.3	15.3 ± 12.8	19.7 ± 24.6	16.9 ± 19.9	19.6 ± 24.0
PGE2	1.5 ± 4.2*	0.2 ± 0.2	1.1 ± 2.4	0.7 ± 2.9	2.1 ± 5.1	0.3 ± 0.2
LTB4	2.3 ± 7.7*	0.3 ± 0.2	3.5 ± 9.9	0.8 ± 4.0	4.4 ± 10.8*	0.3 ± 0.3
13-HODE	16.0 ± 25.5	10.4 ± 6.4	21.3 ± 32.3	11.4 ± 13.3	22.3 ± 32.4*	9.7 ± 4.6
9-HODE	16.7 ± 46.5	7.7 ± 5.1	22.8 ± 54.1	9.6 ± 24.9	25.2 ± 59.7	6.9 ± 3.8
15-HETE	5.5 ± 10.9*	2.1 ± 1.7	6.9 ± 15.8	3.0 ± 4.7	6.8 ± 14.0	2.5 ± 2.0
14sHDHA	36.9 ± 34.6	28.8 ± 27.4	32.4 ± 26.9	33.2 ± 32.3	38.3 ± 31.1	31.2 ± 31.6
11-HETE	4.3 ± 13.8	0.9 ± 0.9	5.8 ± 18.4	1.8 ± 6.5	6.2 ± 17.8	1.1 ± 1.3
12-HETE	200.6 ± 208.9*	119.6 ± 117.8	165.5 ± 159.7	157.0 ± 173.0	192.2 ± 222.2	146.7 ± 148.7
5-HETE	48.5 ± 192.7	1.7 ± 1.2	70.2 ± 265.5	13.6 ± 85.2	71.3 ± 248.0	5.0 ± 19.9
5 oxo- HETE	2.0 ± 7.2	0.2 ± 0.1	2.7 ± 8.2	0.8 ± 4.2	2.9 ± 8.8	0.2 ± 0.2

Values are *n*(%) or mean ± SD. *p < 0.05. *SAD* small airways disease, *ILD* interstitial lung disease

^#^Missing values for PON1 genotype (2 in SAD present, 1 in ILD absent, 1 in bronchiectasis present)

In multivariate regression analyses controlling for other risk factors for lung disease including RA disease duration, age, sex, smoking, and BMI, lower PON1 activity measured by both lactonase and arylesterase assays remained independently associated with the presence of SAD on longitudinal CT scan (Table 3).

**Table 3:** Adjusted multivariate logistic regression analysis of predictors of small airways disease

Predictor	Odds ratio (95% CI)	*P* value	Standard error
Arylesterase	0.99 (0.99–0.99)	0.03	0.00
Lactonase	0.95 (0.90–1.00)	0.04	0.03
Paraoxonase	1.00 (1.00–1.00)	0.67	0.00
PGE2	10.8 (1.47–80.02)	0.02	1.02
LTB4	10.13 (1.10–92.88)	0.04	1.13
12-HETE	1.95 (0.82–4.64)	0.13	0.44
15-HETE	5.17 (1.50–17.78)	0.01	0.63

Adjusted multivariate model includes age at CT scan (years), RA disease duration at CT scan (years), gender (female), BMI (kg/m^2^), and current smoking. Estimate is in U/ml for arylesterase, lactonase, and paraoxonase, and ng/ml for PGE2, LTB4, 12-HETE, and 15-HETE. Model used original normally distributed values for arylesterase, lactonase, and paraoxonase, and log transformation for PGE2, LTB4, 12-HETE, 15-HETE. *CI* confidence intervals

No associations of PON1 or other lipid or disease measures with the presence of ILD or bronchiectasis on longitudinal CT scans were noted, although the percentages of patients with ILD (14.8%), or bronchiectasis (25.0%) were substantially lower than the percentage of patients with SAD (44.4%).

### Higher baseline pro-inflammatory lipid mediators associate with suppressed PON1 activity and the presence of small airways disease on longitudinal follow-up

Baseline levels of pro-inflammatory BLMs, including PGE2, LTB4, 12-HETE, and 15-HETE, were significantly higher in patients with SAD compared to patients without SAD on longitudinal CT scans (Table 2). In multivariate regression analyses controlling for other risk factors for lung disease including RA disease duration, age, sex, smoking, and BMI, higher levels of PGE2, LTB4, and 15-HETE remained independently associated with the presence of SAD on longitudinal follow-up (Table 3). Lower baseline PON1 activity measured by the arylesterase assay correlated significantly with higher levels of both 12-HETE (*r* =  − 0.23, *p* = 0.02), and 15-HETE (*r* =  − 0.21, *p* = 0.04), and lower PON1 activity by the paraoxonase assay correlated significantly with higher levels of 12-HETE (*r* =  − 0.20, *p* = 0.04).

Baseline BLM and PON1 activities did not associate with the presence of ILD on longitudinal CT scans (Table 2). Trends for an association of lower baseline PON1 activities (p > 0.05) and significantly higher levels of LTB4, and 13-HODE (*p* < 0.05) were noted in patients with bronchiectasis compared to patients without bronchiectasis on longitudinal CT (Table 2). LTB4 and 13-HODE levels remained independently associated with the presence of bronchiectasis in multivariate regression adjusting for RA-LD risk factors as above (Supplemental Table 3).

## Discussion

In this retrospective, longitudinal cohort study of RA patients who underwent clinically indicated CT scans of the chest, the majority of patients with RA-LD had radiographic evidence of SAD (44.4%) and smaller numbers of patients demonstrated radiographic evidence of bronchiectasis (25.0%) and ILD (14.8%). Lower baseline PON1 activity measured by both arylesterase and lactonase functions and higher levels of the oxylipins PGE2, LTB4, and 15-HETE were significant predictors of SAD on longitudinal CT after controlling for other known RA-LD risk factors such as smoking, obesity, and RA disease duration. The paraoxonase activity of PON1 did not associate with SAD in this work, suggesting that the relationship between PON1 and SAD may be specific to the arylesterase and lactonase functions of the enzyme.

RA-LD, in particular ILD, shares several traditional risk factors with RA-associated cardiovascular disease including male gender, age, disease duration, obesity, and glucocorticoid use [2, 3]. Our previous work has linked suppressed PON1 activity to both subclinical atherosclerosis measured by carotid plaque [7], as well as risk of cardiovascular events in a large, ~ 2000 patient developmental program of the RA therapeutic, tofacitinib [24]. Due to these shared traditional risk factors for lung and cardiovascular co-morbidities in RA suggesting possible shared pathogenic mechanisms, we pursued the current analysis of the HDL-associated protein, PON1, in RA-LD.

PON1 is a serum enzyme primarily synthesized in the liver and secreted into the plasma where it associates with HDL. PON1 has multiple functions including the metabolism of pro-inflammatory, oxidized lipids in LDL and HDL, and has been implicated in the pathogenesis of several disorders including diabetes, coronary heart disease (CHD), cancer, and lung disease [5, 27]. Our work in the K/BxN mouse model of RA recently showed that over-expression of the human PON1 transgene, which causes a twofold to threefold increase in circulating PON1 activity, reduces RA joint disease activity and associated with lower levels of the pro-inflammatory oxylipins 5-HETE and 15-HETE [26].

The role of PON1 in lung disease remains largely unclear, although some studies have suggested a possible protective role in chronic obstructive lung disease, and inflammatory pulmonary disease related to mustard gas exposure [27, 28]. The PON1 protein has been identified in non-ciliated bronchiolar epithelial cells (clara cells), which maintain the epithelium of the distal conducting airways of the lungs [29], and both the arylesterase activity and protein levels of PON1 have been identified in induced sputum from healthy volunteers [30]. In the work by Chen and colleagues, PON1 over-expression was protective in a murine model of asthma, decreasing bronchial wall thickness and fibrosis, and reducing inflammatory cells in bronchioalveolar lavage fluid [31]. Chen et al. also reported that PON1 inhibited macrophage inflammatory response and fibroblast proliferation in vitro [31].

In the current work, lower PON1 activity associated with higher levels of 12-HETE and 15-HETE, and these pro-inflammatory lipid mediators as well as PGE2, and LTB4 also independently predicted the presence of SAD on longitudinal CT scan. Systemic oxidative stress is implicated in the pathogenesis of RA, and recent work by Polinski et al. has linked the oxylipins 5-HETE and 15-HETE to the development of future incident inflammatory arthritis in an anticitrullinated protein antibody-positive population [32]. These findings suggest PON1 and BLMs as possible novel mechanistic pathways to explore in RA-LD, and further work may be warranted to evaluate the role of PON1 as an alternative therapeutic target in RA and RA-LD. In regards to RA-SAD, the clinical presentation and prognosis is quite varied, from asymptomatic disease, to bronchiolitis obliterans, and better clinical risk stratification tools will be needed in considering who would benefit from therapy. We hope to develop a prospective cohort to better understand the clinical trajectory of RA-SAD to address these important questions.

The relationship between TNFi use and RA-ILD is one of some controversy, and in our work, we did find significantly higher TNFi use at baseline in those with ILD. There is some suggestion in the literature that TNFi are associated with ILD, although these studies are often observational, and cannot confidently differentiate whether this association is causative, versus reflecting underlying disease activity requiring TNFi [33].There is some data that TNFis may show more association with RA-ILD than non-TNFi biologics, which does raise some class-specific concern [34, 35]. Prospective studies to address this question would be highly valuable to minimize observational bias to answer this question.

There is evidence that RA treatments themselves may alter PON1 and BLM levels and activity, although it is hard to interpret how much is from reduced inflammation and RA disease activity, versus medication-specific effects [36–38]. Of note, in our bivariate analysis within treatment groups (methotrexate, hydroxychloroquine, leflunomide, prednisone, TNFi, non-TNFi biologics), there was no statistically significant differences in PON1 activity noted (Supplemental Table 4). So, while there is possibly some contribution from medication use to PON1 activity, our data does not suggest it is a significant contributor in our cohort.

There are limitations to the current work. Because this study was conducted as a retrospective cohort analysis, the lung findings described are of patients who had CT scans of the chest obtained for clinical purposes, which may cause sample bias. Additionally, the CT scans obtained were heterogenous in technique (patient positioning, contrast), and interpreting radiologist. Importantly, the assessment of PON1 activity and bioactive lipid mediators was done approximately 5 years prior to the CT scan and therefore does not reflect the patient’s PON1 activity and levels of oxidative stress at the time the pulmonary disease was identified. Other limitations of the analysis include confounders such as baseline and current medication use, environmental toxins and pollution exposures, socioeconomic status, seropositivity, and erosive status. We did include several covariates which are known to contribute to the development of RA-LD (age, gender, disease duration, smoking, BMI). Baseline medications were not included as we were not able to associate the presence of RA-LD on the readily available CT scan, with the timing of baseline medication use. Additionally, the role of different DMARDs in the development of RA-LD is not yet well elucidated as discussed previously.

In summary, to our knowledge, this is the first study to describe low PON1 activity and elevation in the pro-inflammatory lipid mediators PGE2, LTB4, and 15-HETE as predictors of RA-SAD in longitudinal follow-up. Further work is warranted to confirm these findings and further define the role of PON1 and lipid oxidation in RA-LD.

### Supplementary Information

Below is the link to the electronic supplementary material.

Supplementary file1 (DOCX 17 KB)

Supplementary file2 (DOCX 15 KB)

Supplementary file3 (DOCX 14 KB)

Supplementary file4 (DOCX 13 KB)

Supplementary file5 (DOCX 16 KB)
