# Correction to: Suppressed paraoxonase-1 activity associates with elevated oxylipins and the presence of small airways disease in patients with rheumatoid arthritis

**DOI:** 10.1007/s10067-023-06768-5

**Published:** 2023-09-14

**Authors:** Amir A. Razmjou, Jennifer M. Wang, Ani Shahbazian, Srinivasa Reddy, Christina Charles-Schoeman

**Affiliations:** David Geffen School of Medicine, University of California, Los Angeles, 1000 Veteran Ave, Room 31-79, Los Angeles, CA 90095-1670 USA

**Correction to: Clinical Rheumatology (2022) 42:75-82**

10.1007/s10067-022-06375-w

The final paragraph of the Introduction section as shown below should have been removed in the original published version of the above article.

- “Musculoskeletal ultrasound (MSUS) can detect inflammation within the synovium the joint, and quantification of active synovitis through Power Doppler (PDUS) in RA patients has been well established. PDUS has been utilized to predict RA therapeutic response, RA flares, and clinical remission; and may be more sensitive than clinical examination in detecting active joint inflammation. In the current work, we evaluated an association of PDUS synovitis signal with HDL function and structure in patients with RA treated in two independent clinical therapeutic trials.”

The original article has been corrected.

