# Supplementary material for: Suppressed paraoxonase-1 activity associates with elevated oxylipins and the presence of small airways disease in patients with rheumatoid arthritis
Source: Clin Rheumatol. 2022 Sep 22;42(1):75–82. doi: 10.1007/s10067-022-06375-w (PMC9823017; doi:10.1007/s10067-022-06375-w)
Supplement: Supplementary file 1 — Supplementary file1 (DOCX 17 KB) [file 10067_2022_6375_MOESM1_ESM.docx]

**Supplemental Figure 1.** Types and distribution of radiographic lung disease diagnosed by HRCT chest in a longitudinal RA cohort. N=108 for all cohort patients with CT scans. Small airways disease in 44.4%, interstitial lung disease in 14.8%, and bronchiectasis in 25.0%.
