# Supplementary material for: Suppressed paraoxonase-1 activity associates with elevated oxylipins and the presence of small airways disease in patients with rheumatoid arthritis
Source: Clin Rheumatol. 2022 Sep 22;42(1):75–82. doi: 10.1007/s10067-022-06375-w (PMC9823017; doi:10.1007/s10067-022-06375-w)
Supplement: Supplementary file 2 — Supplementary file2 (DOCX 15 KB) [file 10067_2022_6375_MOESM2_ESM.docx]

Supplemental Table 1: Clinical Characteristics of RA Patients with HRCT Scans for Analysis

| Patient Characteristics (N=108) | |
| --- | --- |
| Age (years) | 63+12.4 |
| Female | 92(85.2) |
| Race- Caucasian | 83(76.9) |
| Ethnicity- Hispanic | 24(22.2) |
| BMI (kg/m^2^) | 29.0+7.4 |
| RA disease duration prior to CT (years) | 19.7+15.0 |
| hsCRP (mg/L) | 8.5+16.9 |
| ESR (mm/hour) | 28.0+21.2 |
| DAS28 | 4.7+1.7 |
| HAQ-DI | 1.1+0.8 |
| **Medications** | |
| Methotrexate | 50(48.1) |
| TNF inhibitor | 43(41.3) |
| Other biologic | 11(11.0) |
| Prednisone | 34(32.7) |
| Statins | 14(13.5) |
| **Cardiovascular Risk Factors** | |
| H/o Myocardial Infarction | 4(4.2) |
| H/o Cerebrovascular Accident | 4(4.3) |
| Hypertension | 52(48.1) |
| Diabetes | 13(12.0) |
| Current tobacco use | 5(5.7) |
| Past tobacco use | 22(26.9) |
| Total Cholesterol (mg/dL) | 194+45.6 |
| LDL Cholesterol (mg/dL) | 108+38.5 |
| HDL Cholesterol (mg/dL) | 63+21.4 |
| Triglycerides (mg/dL) | 126+88.8 |
| Time from Specimen Collection to CT (years) | 5.9+3.5 |

Values are n(%) or mean+SD
