# Supplementary material for: Suppressed paraoxonase-1 activity associates with elevated oxylipins and the presence of small airways disease in patients with rheumatoid arthritis
Source: Clin Rheumatol. 2022 Sep 22;42(1):75–82. doi: 10.1007/s10067-022-06375-w (PMC9823017; doi:10.1007/s10067-022-06375-w)
Supplement: Supplementary file 3 — Supplementary file3 (DOCX 14 KB) [file 10067_2022_6375_MOESM3_ESM.docx]

Supplemental Table 2: Pulmonary Function Tests by Presence of Small Airways Disease

|  | All Patients | Small Airways Disease Present | Small Airways Disease Absent |
| --- | --- | --- | --- |
| FEV1% (n=67) | 85.6(21.2) | 83.8(20.0) | 88.1(22.8) |
| FVC% (n=65) | 89.6(19.1) | 90.5(16.3) | 88.4(22.7) |
| FEV1/FVC (n=66) | 74.1(10.8) | **71.4(12.4)*** | 77.6(7.1) |
| DLCO% (n=62) | 77.0(21.2) | 79.5(15.5) | 73.4(27.2) |
| FEF25-75% (n=65) | 78.1(36.5) | 70.6(34.6) | 88.1(37.3) |

*p<0.05; FEV1/FVC was significantly lower in those with SAD

FEV1: forced expiratory volume in 1 second, FVC: forced vital capacity, DLCO: diffusing capacity of lungs for carbon monoxide, FEF: forced expiratory flow.
