# Supplementary material for: Suppressed paraoxonase-1 activity associates with elevated oxylipins and the presence of small airways disease in patients with rheumatoid arthritis
Source: Clin Rheumatol. 2022 Sep 22;42(1):75–82. doi: 10.1007/s10067-022-06375-w (PMC9823017; doi:10.1007/s10067-022-06375-w)
Supplement: Supplementary file 4 — Supplementary file4 (DOCX 13 KB) [file 10067_2022_6375_MOESM4_ESM.docx]

Supplemental Table 3: Adjusted Multivariate Logistic Regression Analysis of Predictors of Bronchiectasis

| Predictor | Odds Ratio (95% CI) | P value | Standard Error |
| --- | --- | --- | --- |
| LTB4 | 32.43 (1.52-693.36) | 0.03 | 1.56 |
| 13-HODE | 90.49 (5.76-1422.36) | 0.001 | 1.41 |

Adjusted multivariate model includes: age at CT scan (years), RA disease duration at CT scan (years), gender (female), BMI (kg/m^2^), current smoking. Model used log transformed data. CI= confidence intervals.
