# Supplementary material for: Suppressed paraoxonase-1 activity associates with elevated oxylipins and the presence of small airways disease in patients with rheumatoid arthritis
Source: Clin Rheumatol. 2022 Sep 22;42(1):75–82. doi: 10.1007/s10067-022-06375-w (PMC9823017; doi:10.1007/s10067-022-06375-w)
Supplement: Supplementary file 5 — Supplementary file5 (DOCX 16 KB) [file 10067_2022_6375_MOESM5_ESM.docx]

Supplemental Table 4: PON1 activity across baseline treatment groups.

|  | Arylesterase activity | Lactonase activity | Paraoxonase activity | n |
| --- | --- | --- | --- | --- |
| MTX; yes | 182.9+73.4 | 20.8+10.6 | 396.5+304.0 | 50 |
| MTX; no | 204.0+72.8 | 21.9+11.5 | 518.6+441.3 | 54 |
| HCQ; yes | 192.1+81.1 | 22.5+12.9 | 397.8+235.6 | 16 |
| HCQ; no | 194.1+72.5 | 21.2+10.8 | 471.2+405.9 | 88 |
| SSZ; yes | 209.3+94.7 | 26.0+15.8 | 632.4+285.4 | 7 |
| SSZ; no | 192.7+72.2 | 21.1+10.7 | 447.5+389.0 | 97 |
| Prednisone; yes | 212.7+78.3 | 23.1+12.9 | 473.9+362.6 | 34 |
| Prednisone; no | 184.7+69.8 | 20.5+10.0 | 453.1+397.2 | 70 |
| Leflunomide; yes | 208.2+78.7 | 26.3+12.9 | 341.9+249.0 | 11 |
| Leflunomide; no | 192.1+73.1 | 20.8+10.7 | 473.9+396.3 | 93 |
| TNFi; yes | 179.8+65.2 | 18.9+9.8 | 486.1+393.4 | 43 |
| TNFi; no | 203.8+77.8 | 23.1+11.6 | 441.4+380.5 | 61 |
| Non-TNFi biologic; yes | 221.5+68.5 | 22.5+9.8 | 521.0+339.0 | 11 |
| Non-TNFi biologic; no | 191.1+75.2 | 21.3+11.4 | 461.0+395.3 | 89 |

Values are mean+SD

*p<0.05 with two-sided t-test (none were significantly different between medication yes/no)

PON1 activity units are U/ml

Non-TNFi biologic group includes rituximab, tocilizumab, and abatacept

MTX= methotrexate, HCQ= hydroxychloroquine, SSZ= sulfasalazine, TNFi= Tumor necrosis factor inhibitor
